# Households with a Stunted Child and Obese Mother: Trends and Child Feeding Practices in a Middle-Income Country, 1992–2008

**DOI:** 10.1007/s10995-014-1634-5

**Published:** 2014-12-12

**Authors:** Amina Aitsi-Selmi

**Affiliations:** Department of Epidemiology and Public Health, 1-19, Torrington Place, London, WC1E 6BT UK

**Keywords:** Obesity, Stunting, Epidemiology, Socioeconomic factors, Nutrition, Double burden

## Abstract

Middle-income countries in the intermediate stages of the nutrition transition are facing a complex picture of nutrition-related diseases with child stunting and maternal obesity coexisting within single households (SCOB). A debate exists as to whether SCOB is a true phenomenon or a statistical artefact. In this study, we examine time trends and determinants of SCOB in Egypt and test the hypothesis that increased child sugary snack consumption, and reduced fruit/vegetable consumption (markers of poor dietary diversity) are associated with SCOB. Data on 25,065 mothers and their children from the Egyptian Demographic and Health Surveys from 1992, 1995, 2005 and 2008 are used to examine trends in child stunting, maternal obesity and child–mother household type [normal/non-obese, stunted/non-obese, normal/obese, stunted/obese (SCOB)]. The association of child sugary snack and fruit/vegetable consumption with household type is also examined using multinomial logistic regression adjusting for maternal age, maternal education, child age, breastfeeding, household wealth and urban/rural residence. The prevalence of SCOB increased between the periods 1992/95 and 2005/08 despite reductions in stunting levels. This increase paralleled a rise in maternal obesity. Child sugary snack consumption was associated with higher odds (51 %) of belonging to a SCOB household compared with normal/non-obese households, while fruit/vegetable consumption was associated with lower odds (24 %). The results suggest the existence of a link between the rise in maternal obesity and an increase in SCOB, and an association between child sugary snack consumption and SCOB. Addressing SCOB may require a household—rather than individual-based approach to nutrition.

**Electronic supplementary material:**

The online version of this article (doi:10.1007/s10995-014-1634-5) contains supplementary material, which is available to authorized users.

## Introduction

The world is witnessing an unprecedented epidemic of nutrition-related diseases [1–3]. While under-nutrition remains a significant problem in many countries, an alarming rise in obesity in many of these same countries has been reported, including in Egypt which has one of the highest female obesity prevalence levels in the world [4]. This co-existence of over- and under-nutrition has important public health consequences as child under-nutrition severely limits human potential and life expectancy, while obesity is a risk factor for diabetes and heart disease [5].

Therefore, the coexistence of under- and over-nutrition (the double burden of malnutrition) [6] within the same country and among different members of the same household presents an important challenge for public health systems in low- and middle-income countries (LMICs). Households where a child is stunted and the mother is overweight (SCOWT) have been documented in Asia, Latin America and Africa [7–13] including in emergency settings [14], with a prevalence that varies between 2 % in Bangladesh and 25 % in Guatemala [8]. In a survey of 36 LMICs, Egypt was one of only four countries with a prevalence of SCOWT exceeding 10 % [7].

While the existence of SCOWT is relatively well-documented in Latin America and parts of Asia [9, 10, 12, 13], reports from the Middle East and North Africa (MENA) region are fewer [14]. Where reports exist, they focus on maternal overweight rather than obesity [7–10, 12, 14, 15]. However, households where child stunting coexists with maternal obesity (SCOB) may be prevalent in the MENA region, since stunting remains relatively high [16] while maternal obesity levels are among the highest in the world [4, 17].

Factors associated with SCOWT include working in subsistence agriculture, low levels of maternal education and relative household poverty, although these factors may vary by region and country [8–10]. However, a common aetiology for the coexistence of under- and over-nutrition in the same household is difficult to demonstrate, and a recent study argued that the SCOWT phenomenon is a statistical artefact. It inferred that child stunting and maternal overweight occur together at random, that their aetiologies are different, and that the two forms of malnutrition should continue to be addressed separately from a policy and prevention perspective [15]. Yet, epidemiological evidence suggests a link between maternal and child nutrition as early as gestation, and that maternal nutrition can influence the risk of child stunting and subsequent obesity in adulthood [18] through a number of possible mechanisms including intrauterine metabolic programming [19].

In addition, there may be a role for the changes in household diets in countries undergoing the nutrition transition. In these countries, diets are shifting away from traditional foods based on starch and local fruit and vegetables to Western diets that are higher in fat and sugar and lower in fruit and vegetables [2, 4, 20]. Reports that processed foods and sugary drinks are beginning to replace water and milk in the diets of children are emerging [2, 20]. For example, complementary foods given to wean children from breastfeeding in the Middle East and North Africa, including in Egypt, are increasingly composed of sugary drinks and snacks which lack diversity and micronutrient content while being rich in calories [2, 21, 22].

A recent review of infant feeding practices in 46 LMICs showed that inadequate breastfeeding patterns and the premature introduction of complementary foods were common throughout LMICs [23]. It also argued that the high prevalence of child stunting compared with underweight in these countries is linked to worsening dietary diversity and that nutritional interventions need to improve dietary diversity rather than focus solely on energy intake and weight gain [23]. In summary, dietary changes related to the nutrition transition combine calorie abundance and micronutrient scarcity [2, 4, 24], and this imbalance between calorie content and micronutrient content may be problematic in different ways for adults and children. Yet, little is known of the impact of these large scale dietary changes on foetal development, growth in childhood and subsequent adult anthropometric outcomes.

This study seeks to expand the double burden of malnutrition literature by examining trends in prevalence over time of SCOB in a country undergoing the nutrition transition; and by investigating the association of child feeding factors including the consumption of sugary snacks with SCOB. We hypothesise that higher child sugary snack consumption and lower fruit/vegetable consumption (markers of poor dietary diversity) are associated with SCOB.

## Methods

### Dataset

The Demographic and Health Surveys (DHS) are a worldwide project funded by the United States Agency for International Development. Their main objective is to collect nationally representative demographic and health data on women and young children using a multistage stratified probabilistic sampling design based on a standardised methodology [25]. The DHS are a key source of data for studies on SCOWT [7, 8, 26]. Four Egyptian DHS datasets (1992, 1995, 2005 and 2008) are used to represent two distinct time periods. For the examination of trends, the 1992 and 1995 Egyptian DHS datasets were combined to create time period 1 (1992/95), and the 2005 and 2008 datasets were combined to create time period 2 (2005/08).

### Data Collection

The Egyptian DHS collected dietary information for the first time in 2008, using a dietary diversity questionnaire in a subsample of one in three child–mother pairs. Self-reported information on what a child was given to eat in the previous 24 h was recorded. Women and children were weighed on a digital scale, and their weight was recorded in kilograms, to the nearest 100 g. Height was measured using an anthropometer and recorded to the nearest millimetre [27]. The length of children under 24 months was measured lying on a measuring board (Shorr productions^®^) and standing height was measured for older children and their mothers.

### Participants

The total population that took part in the four surveys was 60,644 women (response rate >95 % across all surveys). Only children under 3 years and their mothers were eligible for anthropometry and the dietary survey (N = 30,768 women and their children). Among these women, 3,485 were pregnant and were excluded. Of the remaining 27,583, 1,250 women (4 %) and 901 children (3 %) had missing anthropometry resulting in 25,065 women (aged 15–49 years) and their children (aged 0–3). These participants included 9,201 women and their children in period 1 (1992/95) and 13,376 in period 2 (2005/08). For the multivariate regression, only the 2008 DHS survey was used as this survey is the only survey to include dietary data. The full dietary sample included 5,954 pairs of women and their children. Of these, 513 pregnant women were removed and 110 (2 %) of the pairs had missing covariate data resulting in a final analytic sample of 5,357 mothers and children.

### Dependent Variable

Childhood stunting was defined as having a height for age below minus two standard deviations from the median height-for-age of the international reference population of the US National Centre for Health Statistics. This definition is accepted by the WHO and the US Centers for Disease Control and Prevention (NCHS/WHO/CDC) [28]. Body mass index (BMI) was calculated as weight/height^2^ (in kg/m^2^) and maternal obesity was defined as having a BMI ≥30. Women who were underweight represented <1 % of the sample and were not excluded.

Four different combinations of child–mother pairings were used: normal child/non-obese mother (normal/non-obese = baseline group); stunted child/non-obese mother (stunted/non-obese), normal child/obese mother (normal/obese), stunted child/obese mother (stunted/obese or SCOB).

### Independent Variables

The focus of the multivariate analysis was on the associations between the child feeding factors and the SCOB households. These factors were whether the child was given a sugary snack (chocolate, biscuits or sweets) in the last 24 h (no/yes) and whether the child was given fruit or vegetables in the last 24 h (no/yes).

### Confounders

To keep the model as simple as possible, only the most important confounders were included. The confounders selected were maternal age, maternal education, child age, breastfeeding of the child, household wealth and area of residence as these factors are likely to be associated with feeding practices and the nutritional indicators. Maternal education level was coded into three categories (1 = no or primary education, 2 = secondary education, 3 = higher education). The wealth variable was included in the dataset and places individuals in relative position to each other on a locally appropriate continuous scale of economic status [29]. Data on the ownership of durable assets such as electrical equipment (e.g. TV, computer), basic amenities (e.g. sanitation, water supply) and housing characteristics (e.g. floor material) are used to generate a wealth index score through principal components analysis based on the Filmer and Pritchett method [29]. The component that explains most of the variance of the indicator variables is selected to derive a score for each household, and is divided into quintiles (1 = poorest; 5 = richest) which allows for comparison across countries and studies. Area of residence (urban/rural) was based on administrative criteria used in the DHS sampling strategy [25].

### Statistical Analysis

The STATA version 12SE^®^ (StataCorp., College Station, TX) survey commands (svy) were used when computing the frequencies to account for the complex survey design and the effect of clustering and unequal weights. The prevalence levels in the two time periods of child stunting and maternal obesity were calculated, and a Chi squared test that takes the complex design into account was used to compare the differences between the two periods [30]. To examine the associations between the factors included in the model and each of child stunting, maternal obesity and SCOB, the prevalence of these indicators was calculated for the categorical factors, and the median and interquartile range for cases/non-cases was calculated for the continuous factors. Then, multinomial logistic regression was used to analyse the associations between the independent factors and the outcome indicators using the normal/non-obese pairs as the baseline group. All the odds ratios (ORs) were estimated using the sample from the 2008 DHS (Total N = 5,357). Multicollinearity was examined using individual and mean variance inflation factors.

## Ethical Review

Demographic and Health Surveys (DHS) data collection procedures were approved by the Measure DHS (Calverton, MC) Institutional Review Board and by the national body that approves research studies on humans in Egypt. Written consent was obtained by the interviewers from each participant. The use of the DHS data for this particular study was approved by Measure DHS, and considered exempt from full review by University College London because the study is based on an anonymous, public-use dataset.

## Results

**Fig. 1:**
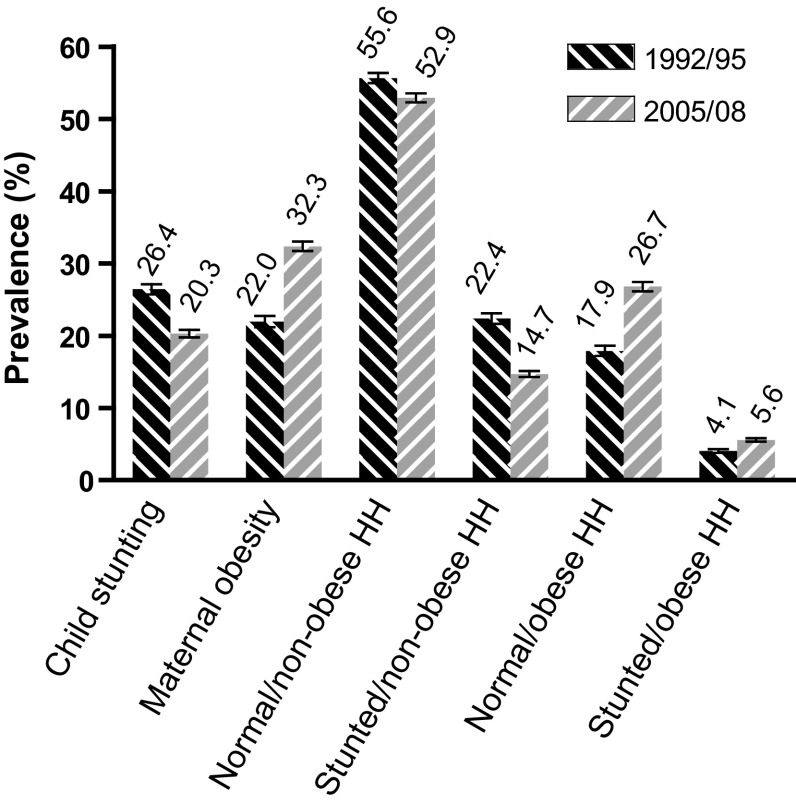
Trends in childhood stunting, maternal obesity and household types (child/mother), Egyptian DHS 1992/92 (N = 9201) and DHS 2005/08 (N = 13,376). *HH* household. *Error bars* represent 95 % confidence intervals. The absolute difference in prevalence between the 1992/95 and 2005/08 time periods was statistically significant for all indicators (*P* < 0.001 for the Chi square test)

A total of 25,065 child–mother pairs were included in the trend analysis of which 94 % represented a unique household. Figure 1 illustrates the difference in prevalence of household types alongside maternal obesity and child stunting between 1992/95 and 2005/08. A decrease in child stunting (26.4–20.3 %) was found and was mirrored by a decrease in stunted/non-obese households. Conversely, an increase in maternal obesity (22.0–32.3 %) was also found and was mirrored by an increase in normal/obese households and SCOB households. The normal/non-obese households also decreased in prevalence over this period. The rest of the results refer to the dietary sample from the 2008 DHS (N = 5,357).

The descriptive statistics in Table 1 show that almost a quarter of all households were in the normal/obese category; and 6.7 % were in the SCOB category. The majority of households (61.8 %) were rural, and the proportion of children receiving a sugary snack (40.0 %) was higher compared with the proportion of children given fruit/vegetable (26.2 %) in the preceding 24 h.

**Table 1:** Summary descriptive statistics, Egypt DHS 2008 (N = 5,357)

N = 5,357	% or median (interquartile range)
Dependent variable	
Household type (child/mother)	
Normal/non-obese	53.8
Stunted/non-obese	14.8
Normal/obese	24.7
Stunted/obese (SCOB)	6.7
Independent variables	
Child given sugary snack in the last 24 h	
Yes	40.0
Child given fruit/vegetable in the last 24 h	
Yes	26.2
Confounders	
Child’s age (months)	
Median	6 (7)
Child ever breastfed	
Yes	96.1
Mother’s age (years)	
Median	27 (8)
Maternal education	
None/primary	33.2
Secondary	53.0
Higher	13.8
Household wealth quintile	
Poorest	20.0
Poorer	19.0
Middle	21.0
Richer	20.0
Richest	20.0
Area of residence	
Urban	38.2
Rural	61.8

The association between the independent factors and each of child stunting, maternal obesity and obese-stunted (SCOB) households is displayed in Table 2. The prevalence of child stunting, maternal obesity and SCOB was higher in the group of children who had been given a sugary snack. The prevalence of maternal obesity and SCOB households increased with wealth but the opposite was true for stunting. Stunting was more common in rural areas while the prevalence of obesity and SCOB was higher in urban areas.

**Table 2:** Association of stunting, obesity and stunted/obese (SCOB) households with the independent variables and the confounders, Egyptian DHS 2008 (N = 5,357)

	Stunted children (N = 942)	Obese mothers (N = 1,001)	Stunted/obese (SCOB) households (N = 260)
	n (%) or median (interquartile range) for non-stunted/stunted	n (%) or median (interquartile range) for non-obese/obese	n (%) or median (interquartile range) for normal/non-obese/SCOB
Independent variables			
Child given sugary snack in the last 24 h			
No	549 (25.1)	558 (26.3)	142 (6.7)
Yes	393 (30.1)	443 (34.5)	118 (9.8)
Child given fruit/vegetable in the last 24 h			
No	665 (26.4)	731 (29.7)	191 (5.0)
Yes	277 (29.1)	270 (28.3)	69 (7.7)
Confounders			
Child’s age (months)	6 (6)/6 (5)	6 (5)/8 (7)	6 (6)/7 (7)
Child ever breastfed			
No	32 (25.1)	43 (28.8)	11 (8.3)
Yes	910 (27.2)	958 (29.6)	249 (7.9)
Maternal age (years)	29 (8)/28 (7)	28 (7)/30 (8)	28 (7)/31 (8)
Maternal education			
None/primary	379 (26.1)	352 (26.4)	90 (6.6)
Secondary	452 (27.1)	521 (30.9)	127 (8.0)
Higher	111 (30.8)	128 (34.6)	43 (12.1)
Household wealth quintile			
Poorest	230 (27.9)	147 (17.9)	38 (4.9)
Poorer	188 (25.1)	166 (24.1)	46 (7.5)
Middle	195 (23.4)	233 (32.5)	57 (8.0)
Richer	176 (20.1)	219 (33.7)	54 (8.5)
Richest	153 (18.3)	236 (42.1)	65 (11.4)
Area of residence			
Urban	321 (25.5)	433 (36.0)	108 (9.2)
Rural	621 (28.0)	568 (22.6)	152 (7.1)

Table 3 presents the adjusted ORs for all the factors in the model and the household types using the normal/non-obese household as the baseline comparison group (the adjusted and unadjusted ORs are presented in the supplemental table). The variance inflation factors (VIFs) were calculated and showed that multicollinearity was not an issue: none of the individual variable VIFs were >10 and the mean VIF was <3.

**Table 3:** Multivariate associations for the three types of household with the different factors, Egypt DHS 2008 (N = 5,357)

N = 5,357	Stunted/non-obese households^a^	Normal/obese households^a^	Stunted/obese (SCOB) households^a^
	Adjusted OR (95 % CI)	Adjusted OR (95 % CI)	Adjusted OR (95 % CI)
Independent variables			
Child given sugary snack			
No (Ref.)	1	1	1
Yes	1.32 (1.12–1.55)***	1.33 (1.12–1.57)***	1.51 (1.17–1.94)***
Child given fruit/vegetables			
No (Ref.)	1	1	1
Yes	1.14 (0.99–1.39)	0.79 (0.66–0.96)*	0.76 (0.57–0.97)*
Confounders			
Child’s age	1.02 (1.00–1.05)	1.05 (1.03–1.08)**	1.00 (1.00–1.01)*
Child ever breastfed			
No (Ref.)	1	1	1
Yes	0.79 (0.69–1.11)	0.83 (0.56–1.22)	0.84 (0.64–1.11)
Maternal age	0.99 (0.98–1.00)	1.0 (1.02–1.07)***	1.08 (1.04–1.16)***
Maternal education	0.95 (0.89–0.98)*	0.98 (0.92–1.05)	1.02 (0.80–1.27)
Household wealth	0.93 (0.89–0.97)*	1.32 (1.22–1.42)***	1.38 (1.23–1.56)***
Area of residence			
Urban (Ref.)	1	1	1
Rural	1.09 (1.01–1.12)*	0.89 (0.83–0.96)*	0.95 (0.83–1.25)

*Ref.* reference category

* *P* < 0.05; ** *P* < 0.01; *** *P* < 0.001

^a^Estimates from the multinomial logistic regression using normal/normal households as the baseline category

Education and wealth were included as continuous variables because tests for linearity showed that assuming a linear relationship was reasonable (full results available on request). Sugary snack consumption was positively associated with all the household types increasing the odds of belonging to these by 32, 33 and 51 % (adjusted OR: 1.32; 95 % CI 1.12–1.55; 1.33; 1.12–1.57; 1.51; 1.17–1.94 for stunted/non-obese, normal/obese and SCOB households compared with normal/non-obese households respectively). An association between fruit/vegetable consumption and the odds of belonging to a normal/obese or a SCOB household compared with a normal/non-obese household was also found, with the odds being lower by 21 and 24 % respectively (see Table 3). Of note, the association between breastfeeding and the household types was not statistically significant but the magnitude of the ORs suggested that breastfeeding decreased the odds of belonging to a household with a stunted child, obese mother or a SCOB household compared with a normal/non-obese household. The odds of belonging to a stunted/non-obese household were lower in wealthier households by 7 % for each wealth quintile, while the odds of belonging to a normal/obese or SCOB household were higher by 32 and 38 % respectively for each quintile of wealth compared with a normal/non-obese household.

### Sensitivity Analysis

Other potential confounders were explored in the analysis. These confounders were birth parity (number of children ever born to the mother) which is associated with a greater risk of excess maternal weight [31], child sex and birth order as these factors influence the risk of stunting, whereby boys and children of higher birth order are at greater risk [22, 32], as well as household size. These factors did not have an impact on the magnitude of the estimates or their statistical significance.

## Discussion

This study examines trends in SCOB and its association with child feeding factors in Egypt. We find that the prevalence of SCOB households in Egypt has increased from 4.1 to 5.6 % between 1992/95 and 2005/2008 mirroring an increase in maternal obesity from 22.0 to 32.3 % in this sample. We also find that child feeding practices related to the nutrition transition (higher sugary snack consumption and lower fruit/vegetable consumption) increase the likelihood of belonging to a SCOB household.

The findings add to previous studies reporting significant levels of child stunting and maternal overweight/obesity [4, 7]. This study shows that Egypt has seen relatively modest declines in stunting (26.4–20.3 %) while obesity levels have risen significantly (22.0–32.3 %) over the period examined. The implication is that improvements in stunting levels may be offset by a rise in maternal obesity resulting in a rise in the double burden of malnutrition including SCOWT/SCOB households.

The associations found between child feeding factors and SCOB could be explained by chance such that no common aetiology to child stunting and maternal obesity truly exists [15]. However, the body of literature on the nutrition transition and the double burden of malnutrition points to a role for changing diets. A household diet that favours energy-dense food and that is poor in fruit and vegetables is unlikely to be adequate for child growth and adult health, whereby a higher energy intake alone is probably ineffective in preventing child stunting and may increase the risk of obesity in adult women. In other words, the quality of a household diet in terms of diversity and micronutrient content may be as important as calorie provision, as has been argued elsewhere [23].

Other studies that support such an explanation include a refugee camp survey in Algeria [14]. The study documents high rates of child stunting and maternal obesity in this emergency setting where the population has become reliant on food assistance packages that lack diversity and are rich in sugars and poor in fruit and vegetables [14]. Parallels can be drawn with the dietary situation in Egypt where the national diet is influenced by a long-standing food subsidy on sugar, bread and oil but not on fruit or vegetables [33]. Furthermore, the consumption of soft drinks in Egypt has increased dramatically over the period examined in this study, including in poorer groups [34]. Therefore, the calorie-rich nature of the Egyptian diet, worsened by the nutrition transition, may encourage both child stunting and maternal obesity and explain the relatively high levels of child stunting, maternal obesity and SCOB in this country. The characterisation of pathways linking these dietary changes to SCOB through foetal development, growth in childhood and subsequent adult metabolic outcomes requires further research.

Of note, SCOB households were more likely to be wealthy in this study, but other studies report different socioeconomic patterns for households displaying both under- and over-nutrition among its members. In a study from Guatemala, SCOWT was more common in the poor and middle-income groups, while in a study from Bangladesh, the prevalence was higher in households with higher expenditure. A recent review of SCOWT in 18 LMICs using DHS datasets did not find any clear association with wealth suggesting that there may be significant country variation [8, 10]. The association between SCOWT and urban/rural residence also appears to differ by country [7, 8, 10]. The heterogeneity in these associations may be related to country differences in dietary and cultural factors, overall educational and income levels and the social distribution of obesity which are all known to change over time as countries develop [35–37]. Therefore, the socioeconomic determinants of SCOB are not straightforward and need to be identified on a country by country basis.

### Implications for Health Services and Policy Makers

Few policies or programmes engage with the need to address both over- and under-nutrition simultaneously [6]. Yet, the solution need not be complex, as nutritional advice can be based on recommendations that support healthy nutrition in childhood and adulthood. Recommendations could promote dietary diversity through reducing the consumption of energy-dense, low micronutrient foods including processed foods, and increasing the consumption of fruit and vegetables in countries undergoing the nutrition transition. In Egypt, such recommendations may be particularly beneficial in countering the effects of the premature introduction of complementary foods including sweetened water and biscuits that occurs across the income spectrum [21], as well as addressing the historical overreliance on a food subsidy system that favours sugars and oil. Health professionals working in settings where SCOB exists could improve the effectiveness of nutritional interventions by taking a household-based rather than individual-based approach when conducting nutritional assessments and giving nutritional advice.

### Strengths and Limitations

The cross-sectional nature of the data limits causal inference in this study, and dietary variables are known to be subject to reporting bias. However, we found strong associations between feeding patterns and SCOB that support the hypothesis that sugary snacks and low fruit/vegetable consumption may be associated with SCOB and contribute to a common aetiology for child stunting and maternal obesity. Further research could include a broader range of dietary indicators or a composite indicator to capture dietary diversity; investigate the biological and metabolic pathways linking diet and SCOB; disaggregate the analysis by urban/rural residence to investigate the socioeconomic distribution of SCOB in more detail; as well as examine the role of knowledge and attitudes towards nutrition.

## Conclusion

The findings from this study support a broad definition of malnutrition as a multifaceted phenomenon that encompasses both under- and over-nutrition in countries undergoing the nutrition transition. They also indicate that dietary changes related to the nutrition transition may have different, negative impacts on children and women. While there have been improvements in child stunting levels in Egypt, the gains appear to have been offset by rising maternal obesity levels, resulting in an increase in stunted child/obese mother (SCOB) households. We recommend a public health approach that addresses poor nutrition as a single phenomenon and encourage health professionals to provide nutritional advice targeting households rather than individuals, basing their advice on recommendations that improve dietary diversity and not energy intake alone.

## Electronic supplementary material

Below is the link to the electronic supplementary material.

Supplementary material 1 (DOCX 18 kb)

## References

[1] Popkin BM (2009). What can public health nutritionists do to curb the epidemic of nutrition-related noncommunicable disease?. Nutrition Reviews.

[2] Popkin BM, Adair LS, Ng SW (2012). Global nutrition transition and the pandemic of obesity in developing countries. Nutrition Reviews.

[3] Finucane MM, Stevens GA, Cowan MJ (2011). National, regional, and global trends in body-mass index since 1980: Systematic analysis of health examination surveys and epidemiological studies with 960 country-years and 9.1 million participants. Lancet.

[4] Ng M, Fleming T, Robinson M (2014). Global, regional, and national prevalence of overweight and obesity in children and adults during 1980–2013: A systematic analysis for the Global Burden of Disease Study 2013. Lancet.

[5] Uauy R, Kain J, Mericq V (2008). Nutrition, child growth, and chronic disease prevention. Annals of Medicine.

[6] Shrimpton, R., & Rokx, C. (2012). *The double burden of malnutrition: A review of the global evidence*. Health, Nutrition and Population (HNP) Discussion Paper. Washington, DC: The International Bank for Reconstruction and Development/The World Bank.

[7] Garrett J, Ruel MT (2005). The coexistence of child undernutrition and maternal overweight: Prevalence, hypotheses, and programme and policy implications. Maternal & Child Nutrition.

[8] Jehn M, Brewis A (2009). Paradoxical malnutrition in mother-child pairs: Untangling the phenomenon of over- and under-nutrition in underdeveloped economies. Economics & Human Biology.

[9] Lee J, Houser RF, Must A (2012). Socioeconomic disparities and the familial coexistence of child stunting and maternal overweight in Guatemala. Economics & human Biology.

[10] Oddo VM, Rah JH, Semba RD (2012). Predictors of maternal and child double burden of malnutrition in rural Indonesia and Bangladesh. American Journal of Clinical Nutrition.

[11] Roemling C, Qaim M (2013). Dual burden households and intra-household nutritional inequality in Indonesia. Economics & Human Biology.

[12] Doak CM, Adair LS, Monteiro C (2000). Overweight and underweight coexist within households in Brazil, China and Russia. The Journal of Nutrition.

[13] Bassete MN, Romaguera D, Gimenez MA (2014). Prevalence and determinants of the dual burden of malnutrition at the household level in Puna and Quebrada of Humahuaca, Jujuy, Argentina. Nutricion Hospitalaria.

[14] Grijalva-Eternod CS, Wells JC, Cortina-Borja M (2012). The double burden of obesity and malnutrition in a protracted emergency setting: A cross-sectional study of Western Sahara refugees. PLoS Medicine.

[15] Dieffenbach S, Stein AD (2012). Stunted child/overweight mother pairs represent a statistical artifact, not a distinct entity. Journal of Nutrition.

[16] de Onis M, Blossner M, Borghi E (2012). Prevalence and trends of stunting among pre-school children, 1990–2020. Public Health Nutrition.

[17] Kilpi F, Webber L, Musaigner A (2014). Alarming predictions for obesity and non-communicable diseases in the Middle East. Public Health Nutrition.

[18] Black RE, Allen LH, Bhutta ZA (2008). Maternal and child undernutrition: Global and regional exposures and health consequences. Lancet.

[19] Gluckman PD, Hanson MA, Beedle AS (2008). Fetal and neonatal pathways to obesity. Frontiers of Hormone Research.

[20] Popkin BM (2006). Global nutrition dynamics: The world is shifting rapidly toward a diet linked with noncommunicable diseases. American Journal of Clinical Nutrition.

[21] Nasreddine L, Zeidan MN, Naja F (2012). Complementary feeding in the MENA region: Practices and challenges. Nutrition, Metabolism, and Cardiovascular Diseases.

[22] Zottarelli LK, Sunil TS, Rajaram S (2007). Influence of parental and socioeconomic factors on stunting in children under 5 years in Egypt. Eastern Mediterranean Health Journal.

[23] Lutter CK, Daelmans BM, de Onis M (2011). Undernutrition, poor feeding practices, and low coverage of key nutrition interventions. Pediatrics.

[24] Popkin B, Gordon-Larsen P (2004). The nutrition transition: Worldwide obesity dynamics and their determinants. International Journal of Obesity.

[25] Zanati, F. Egypt Go. Egyptian Demographic and Health Surveys reports (1992, 1995, 2005, 2008). Measure DHS. Available at http://www.measuredhs.com/aboutsurveys/search/listmodules_main.cfm.

[26] Subramanian SV, Kawachi I, Smith GD (2007). Income inequality and the double burden of under- and over-nutrition in India. Journal of Epidemiology and Community Health.

[27] Mukuria, A., Aboulafia, C., & Themme, A. (2005). DHS Comparative Reports. The context of women’s health: Results from the Demographic and Health Surveys 1994–2001. Calverton, USA.

[28] WHO. (1995). Report of the World Health Organization Expert Committee. Physical status: The use and interpretation of anthropometry. Geneva, Switzerland. 8594834

[29] Rutstein, S.O., & Johnson, K. (2004). ORC Macro. MEASURE/DHS+ (Programme). The DHS wealth index. Calverton, Md.: ORC Macro, MEASURE DHS+.

[30] Heeringa S, West BT, Berglund PA (2010). Applied survey data analysis.

[31] Brooks R, Maklakov A (2010). Sex differences in obesity associated with total fertility rate. PLoS ONE.

[32] Ukwuani FA, Suchindran CM (2003). Implications of women’s work for child nutritional status in sub-Saharan Africa: A case study of Nigeria. Social Science & Medicine.

[33] Asfaw A (2007). Micronutrient deficiency and the prevalence of mothers’ overweight/obesity in Egypt. Economics & Human Biology.

[34] Euromonitor. Soft drinks in Egypt (2012). Euromonitor country reports. Available at http://www.euromonitor.com/soft-drinks-in-egypt/report.

[35] Monteiro CA, Conde WL, Lu B (2004). Obesity and inequities in health in the developing world. International Journal of Obesity and Related Metabolic Disorders.

[36] Dinsa GD, Goryakin Y, Fumagalli E (2012). Obesity and socioeconomic status in developing countries: A systematic review. Obesity Reviews.

[37] Aitsi-Selmi A, Bell R, Shipley MJ (2014). Education modifies the association of wealth with obesity in women in middle-income but not low-income countries: An interaction study using seven national datasets, 2005–2010. PLoS ONE.

