# Supplementary material for: Households with a Stunted Child and Obese Mother: Trends and Child Feeding Practices in a Middle-Income Country, 1992–2008
Source: Matern Child Health J. 2014 Dec 12;19(6):1284–91. doi: 10.1007/s10995-014-1634-5 (PMC4445768; doi:10.1007/s10995-014-1634-5)
Supplement: Supplementary file 1 — Supplementary material 1 (DOCX 18 kb) [file 10995_2014_1634_MOESM1_ESM.docx]

**Supplemental table** Bivariate and multivariate associations for the three types of household with the different predictors, Egypt DHS, 2008 (N=5357).

|  | Stunted/non-obese households^1^ | | Normal/obese households^1^ | | Stunted/obese (SCOB) households^1^ | |
| --- | --- | --- | --- | --- | --- | --- |
| N=5357 | Unadjusted  OR (95%CI) | Adjusted  OR (95%CI) | Unadjusted  OR (95%CI) | Adjusted  OR (95%CI) | Unadjusted  OR (95%CI) | Adjusted  OR (95%CI) |
|  |  |  |  |  |  |  |
| **Independent variables** |  |  |  |  |  |  |
| Child given sugary snack |  |  |  |  |  |  |
| No (Ref)^2^ | 1 | 1 | 1 | 1 | 1 | 1 |
| Yes | 1.37 (1.18-1.61)*** | 1.32 (1.12-1.55)*** | 1.46 (1.25-1.70)*** | 1.33 (1.12-1.57)*** | 1.69 (1.34-2.15)*** | 1.51 (1.17-1.94)*** |
|  |  |  |  |  |  |  |
| Child given fruit/vegetables |  |  |  |  |  |  |
| No (Ref)^2^ | 1 | 1 | 1 | 1 | 1 | 1 |
| Yes | 1.27 (1.08-1.50)** | 1.14 (0.99-1.39) | 1.01 (0.84-1.18) | 0.79 (0.66-0.96)* | 1.07 (0.83-1.41) | 0.76 (0.57-0.97)* |
|  |  |  |  |  |  |  |
|  |  |  |  |  |  |  |
| **Confounders** |  |  |  |  |  |  |
| Child's age | 1.01 (0.99-1.03) | 1.02 (1.00-1.05) | 1.08 (1.07-1.10)*** | 1.05 (1.03-1.08)** | 1.02 (0.98-1.06) | 1.00 (1.00-1.01)* |
|  |  |  |  |  |  |  |
| Child ever breastfed |  |  |  |  |  |  |
| No (Ref)^2^ | 1 | 1 | 1 | 1 | 1 | 1 |
| Yes | 1.15 (0.88-1.34) | 0.79 (0.69-1.11) | 0.88 (0.61-1.18) | 0.83 (0.56-1.22) | 0.84 (0.47-1.51) | 0.84 (0.64-1.11) |
|  |  |  |  |  |  |  |
| Maternal age | 1.01 (0.99-1.01) | 0.99 (0.98-1.00) | 1.09 (1.07-1.10)*** | 1.0 (1.02-1.07)*** | 1.09 (1.07-1.12)*** | 1.08 (1.04-1.16)*** |
|  |  |  |  |  |  |  |
| Maternal education | 0.85 (0.71-0.92)*** | 0.95 (0.89-0.98)* | 1.17 (1.04-1.31)*** | 0.98 (0.92-1.05) | 1.29 (1.08-1.55)*** | 1.02 (0.80-1.27) |
|  |  |  |  |  |  |  |
| Household wealth | 0.90 (0.87-0.98)*** | 0.93 (0.89-0.97)* | 1.27 (1.20-1.34)*** | 1.32 (1.22-1.42)*** | 1.31 (1.21-1.43)*** | 1.38 (1.23-1.56)*** |
|  |  |  |  |  |  |  |
| Area of residence |  |  |  |  |  |  |
| Urban (Ref)^2^ | 1 | 1 | 1 | 1 | 1 | 1 |
| Rural | 1.16 (1.07-1.26)* | 1.09 (1.01-1.12)* | 0.66 (0.56-0.77)*** | 0.89 (0.83-0.96)* | 0.76 (0.60-0.97)*** | 0.95 (0.83-1.25) |
|  |  |  |  |  |  |  |

^1^ Estimates from the multinomial logistic regression using normal/normal households as the baseline category

^2^ Ref = reference category

**P* <0.05; ***P*<0.01; ** *P<0.001*
